# Green Route for the Upcycling of Urban Bioplastic Waste: A Biotechnological Resource Recovery Strategy toward Medium-Chain Fatty Acids Production

**DOI:** 10.1021/acsenvironau.6c00074

**Published:** 2026-07-22

**Authors:** Stefania Angelini, Agata Gallipoli, Andrea Gianico, Francesca Angelini, Michela Sbicego, Vincenzo Piemonte, Camilla M. Braguglia

**Affiliations:** † Water Research Institute, 124216 National Research Council (CNR-IRSA), Strada Provinciale 35d, 9, Montelibretti, Rome 00010, Italy; ‡ Department of Chemical Engineering, Materials, Environment, Sapienza - University of Rome, Piazzale Aldo Moro 5, Rome 00185, Italy; § University Campus Bio-Medico of Rome, via Alvaro Del Portillo 21, Rome 00128, Italy

**Keywords:** Thermoplastic Starch, Poly(lactic acid), Upcycling, Anaerobic Fermentation, Chain Elongation, Carboxylic Acids, Additives

## Abstract

Bioplastics are expected to represent an expanding fraction of urban waste. Recycling and upcycling remain still limited, yet bioplastics are suitable feedstocks for producing value-added products. Developing circular valorization pathways is essential to prevent resource loss and environmental degradation. This study presents an integrated chemo-biotechnological strategy to upcycle bioplastic waste toward medium-chain fatty acid production. Hydrothermal pretreatments were optimized to chemically recycle typical domestic items, such as thermoplastic starch-based bags and poly­(lactic acid)-based cups. The yield of carbohydrates extracted from the bag was 86 wt % at 134 °C, 20 min, while the yield of lactic acid from the cup was around 40 wt %. The resulting enriched hydrolysates were utilized in cascade fermentation batch tests using a mixed microbial culture. The system successfully converted the recycled carbohydrates into ethanol, acetate, and butyrate, subsequently leveraging the recycled lactate as an electron donor for anaerobic chain elongation toward medium-chain fatty acids. This process yielded caproate and caprylate concentrations of up to 3000 mgCOD/L and 1300 mgCOD/L, respectively. By decoupling chemical deconstruction from microbial synthesis, this integrated approach enables the recovery of strategic building blocks that are otherwise lost in incineration or landfilling practices. Overall, this study establishes a robust groundwork for transitioning from fossil-dependent chemical production to a waste-based circular bioeconomy, offering a proof of concept to demonstrate the feasibility of upcycling mixed urban bioplastics into platform chemicals.

## Graphical abstract

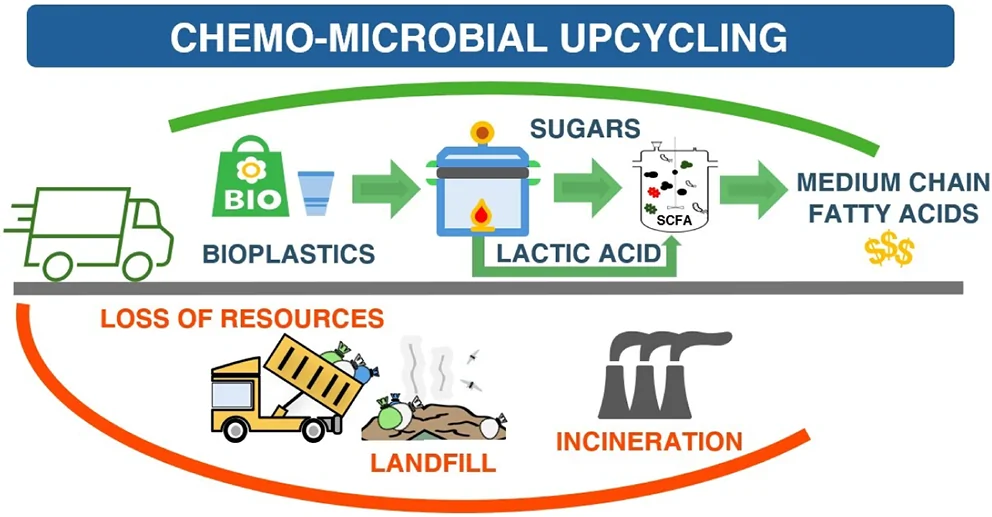

## 1 Introduction

The transition toward a circular economy is driving the adoption of bioplastics as alternatives to conventional plastics.^1^ Bioplastics encompass a broad range of materials that can be bio- or fossil-based and biodegradable or nonbiodegradable, depending on environmental conditions.^2–4^ Among biodegradable bioplastics, poly­(lactic acid) (PLA), thermoplastic starch (TPS), and poly­(hydroxyalkanoates) (PHAs) currently dominate the market.^5,6^ PLA is a biobased aliphatic polyester produced from lactic acid obtained by carbohydrate fermentation,^7^ whereas TPS is derived from starch-rich crops and requires plasticization and subsequent modification or blending to overcome its inherent brittleness and poor processability.^8–11^ In 2024, PLA and TPS represented 37.1% and 5.7% of global biodegradable bioplastics production capacity, respectively.^6^ PLA is mainly used in rigid food-packaging applications, such as coated paper cups and tableware,^12^ while TPS is widely employed in flexible packaging, including carrier and organic-waste collection bags.^7^ With global bioplastics production expected to reach 5.7 Mt by 2029,^6^ increasing quantities of these materials will enter municipal waste streams, creating an urgent need for effective end-of-life (EoL) management strategies. Recent European regulations, including the Recycled Plastics Regulation (EU) 2022/1616 and the Packaging and Packaging Waste Regulation (PPWR), increasingly prioritize recycling over composting for PLA and noncontaminated TPS products.^13–15^ This approach aims to retain carbon within the economy, contribute to recycled-content targets, and avoid the loss of valuable biobased polymers through biodegradation. Consequently, the development of suitable recycling technologies for these materials is becoming increasingly important. Chemical recycling, particularly hydrolysis, is a promising process based on depolymerization of polymeric waste into smaller molecules, which can be then either used to resynthesize the original polymer, with physical–chemical properties comparable to those of the virgin material (i.e., closed-loop recycling), or to achieve other useful materials (i.e., upcycling).^16–19^ Its efficiency depends on material characteristics and operating conditions such as temperature and pH.^20–23^ Previous studies have demonstrated the hydrolysis of TPS-based products^24,25^ and PLA items,^26–36^ achieving the recovery of carbohydrates and lactic acid with high conversion efficiencies. Recovered lactic acid can subsequently serve not only as a feedstock for PLA production but also as a platform molecule for the synthesis of value-added chemicals.^21,37^ Among emerging upcycling strategies, the biological production of medium-chain fatty acids (MCFAs) through chain elongation (CE) is particularly attractive. MCFAs, such as caproic acid with 6 carbon atoms, are valuable chemicals currently produced mainly from fossil resources.^38,39^ In CE, short-chain fatty acids (SCFAs) are biologically elongated using electron donors such as ethanol or lactic acid.^40–42^ Although a few studies have explored the valorization of biodegradable plastics through hydrothermal treatment followed by fermentation^44,45^ or have demonstrated the use of recycled lactic acid as an electron donor for CE,^46^ the biological upcycling of postconsumer bioplastics remains largely unexplored.^43^ Overall, previous research has focused on (i) chemical recycling of PLA or TPS for monomer recovery or chemical upgrading;^24,26,36^ (ii) fermentation of hydrolysates, typically limited to SCFA generation;^44,45^ and (iii) CE processes relying on externally supplied electron donors, with only isolated examples using recycled lactic acid.^46^ However, no study has demonstrated an integrated cascade process starting from real PLA- and TPS-based consumer products in which hydrolysis products are selectively directed toward complementary biological pathways. Therefore, the objective of this study is to demonstrate a system in which TPS-extracted carbohydrates are fermented to SCFAs, while PLA-derived lactate is simultaneously recovered and repurposed as an electron donor to drive anaerobic chain elongation toward MCFAs. The integration of a mild hydrothermal pretreatment as a preparatory step for targeted biological upgrading to recover and convert carbon from the most used bioplastic waste into platform chemicals represents an innovative approach to move toward the development of an integrated biorefinery platform.

## 2 Materials and Methods

### 2.1 Bioplastic and Biomass

TPS-based bag (SBB) and PLA-based cup (PLAc), hereinafter referred to as bioplastics, were purchased from a local supermarket and were labeled as biodegradable and compostable according to UNI EN 13432. SBB and PLAc were cut into squares of approximately 2 cm × 2 cm (Figure 1) and kept at 45 °C in an oven before use.

**Figure 1:**
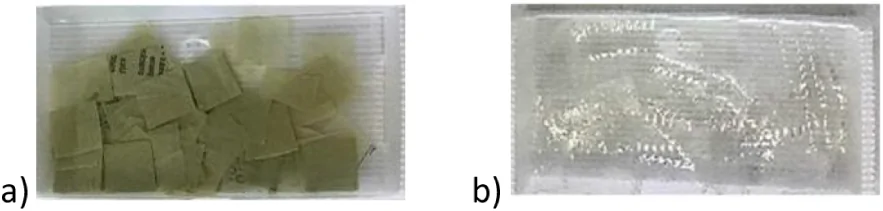
a) SBB bag and b) PLAc cup squared pieces.

The inoculum used for the tests was a mixed-culture biomass, including Actinobacteria and Firmicutes phyla,^47^ highly specialized in fermentative processes. It was collected from a lab-scale anaerobic fermenter treating organic waste and accurately sieved (mesh 1.7 mm) to remove residual waste particles. Phosphate-buffered saline (PBS) solution containing (per L) NaCl 8 g, KCl 0.2 g, Na_2_HPO_4_ 1.44 g, KH_2_PO_4_ 0.25 g was used to cleanse the biomass of carboxylic acids (biomass:PBS 1:1 w/v). The biomass was centrifuged at 4000 rpm for 4 min, repeated 4 times using a Rotanta 460 centrifuge, collecting the supernatant after each cycle. Supernatants were collected after the 4 washing cycles and labeled as supernatant 1, supernatant 2, supernatant 3 and supernatant 4, respectively. Afterward, the residual cleansed biomass was dispersed in a basal medium containing (per L) (NH_4_)­SO_4_ 0.5 g, MgSO_4_ 7H_2_O 0.1 g, CaCl_2_ 2H_2_O 0.05 g, K_2_HPO_4_ 0.11 g, KH_2_PO_4_ 0.51 g, FeCl_3_ 0.1 mL, and Na_2_EDTA 0.1 mL up to 18.5 w/v% concentration. The biomass was then preincubated at 37 °C for 5 days to condition it before its use as inoculum.

### 2.2 Chemicals and Experimental Design

Sulfuric acid (95%) and a 5 wt % phenol solution were used for colloidal carbohydrate determination. A 1 wt % Suprapur HNO_3_ solution was prepared to perform ICP-OES analysis. A 3 M sodium hydroxide solution (NaOH) was prepared to perform PLAc alkaline hydrolysis and to trap CO_2_ in batch-scale fermentative tests. Glucose powder and lactic acid 90% were purchased from VWR International. The d/l-lactic acid assay kit (Megazyme, Ireland) was used to measure spectrophotometrically the concentration of total lactate ions. A 0.33 M oxalic acid solution in Milli-Q water was prepared and used at 10 vol % to acidify samples for carboxylic acid detection. All reagents were of analytical grade. The overall chemical characterization is deeply described in Section 2.6.

The experimental design is illustrated in Figure 2.

**Figure 2:**
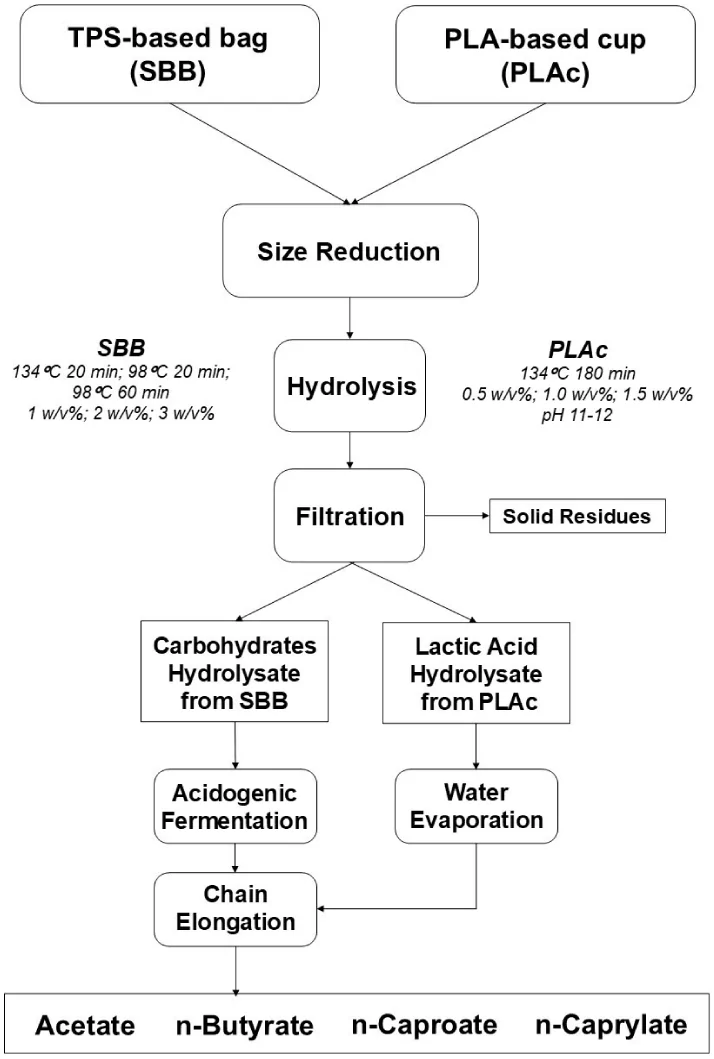
Illustration of experimental design.

### 2.3 Hydrothermal Pretreatment of Bioplastics

A mild hydrothermal pretreatment (HTP) was performed using a bench-scale autoclave Laboklav 25b (SHP Steriltechnik AG, Germany). The tests on SBB were conducted in 1 L bottles with 200 mL of distilled water at bioplastic/water concentrations of 1 w/v%, 2 w/v%, and 3 w/v% in duplicate. Neutral hydrolysis was performed, following the operative constraints of the equipment, at *T* = 98 °C (*p* = 1.8 bar) and *T* = 134 °C (*p* = 3.2 bar) for 20 min and at *T* = 98 °C for 60 min. Alkaline hydrolysis of PLAc was performed at *T* = 134 °C for 180 min, *p* = 3.2 bar, with 0.5 mL of NaOH 3 M/1 L distilled water and bioplastic/water concentrations of 0.5 w/v%, 1.0 w/v%, and 1.5 w/v%. Afterward, bioplastic samples were recovered through gravity-driven cellulose (porosity 10–20 μm) filtration and dried in an oven at 45 °C until constant weight. The disintegration degree of bioplastics was calculated as reported in Angelini et al. (2025)^25^ based on mass reduction. Besides, the aqueous hydrolysates were collected from each HTP condition for further characterization of extracted carbohydrates and lactic acid.

Total lactic acid was assumed to be equal to volatile solids (VS) detected in PLAc, while total carbohydrates were measured as reported in Section 2.6.

The PLAc hydrolysate was acidified with 1% HNO_3_, and the resulting solution was analyzed by ICP-OES (Agilent 5800) to determine the concentration of metals potentially extracted during hydrothermal pretreatment that could affect subsequent cascade processes. The analysis was also performed on a distilled water sample subjected to hydrothermal pretreatment at 134 °C for 20 min. This sample was used as a control to account for and exclude metals originating from the water.

### 2.4 Acidogenic Fermentation Tests

Acidogenic fermentation batch tests were carried out at 37 °C in 0.5 L-reactors by using the Automatic Methane Potential Test System (AMPTS II, Bioprocess Control, Sweden). Each reactor was mechanically stirred and heated at 37 ± 1 °C. N_2_ gas was flushed to ensure an anaerobic environment in the reactor headspaces before sealing. CO_2_-fixing unit vials were filled with 80 mL of 3 M NaOH. Hydrogen content was measured online using an automated data logging system. Biomass was supplied at a fixed concentration of 18.6 ± 0.1 w/v% in basal medium and used as inoculum. A reactor was fed with 175 ± 1 mL of hydrolysate from SBB, treated in an autoclave at 134 °C for 20 min at 1 w/v%, to attain a final carbohydrate concentration of 855 ± 44 mgCOD/L in the reactor. Control reactors with equivalent synthetic glucose concentrations and a blank reactor containing the inoculum alone were established. All tests were prepared in duplicate and lasted 8 days. Hereinafter, the reactors fed with carbohydrates from SBB HTP, glucose, and the one with sole inoculum were referred to as Test, Control, and Blank, respectively. Daily (except on weekends), around 2 mL of broth were withdrawn with a syringe to measure pH and carboxylic acid/ethanol content after filtration through nylon syringe filters (0.22 μm porosity).

### 2.5 Chain Elongation Tests

As an initial step, synthetic lactic acid was added to the Control and Test reactors on day 8 from the start of acidogenic fermentation up to a 1070 ± 4.1 mgCOD/L concentration, in order to assess the occurrence of chain elongation in both systems (Supporting Information). Then, the experimental lactic acid feeding, focus of this investigation, was done after a deliberately extended interval to ensure complete consumption of the initial carbon substrate and to allow the microbial community sufficient time to adapt to chain elongation pathways. Chain elongation tests were performed by adding hydrolysate containing lactic acid from PLAc HTP to the fermentative broths of Test reactors, previously fed with carbohydrates from SBB HTP, up to 1717 ± 30 mgCOD/L and 2798 ± 25 mgCOD/L lactic acid concentrations. Synthetic lactic acid was also tested and added to Control reactors, previously fed with glucose, reaching around 1738 ± 25 mgCOD/L and 2780 ± 18 mgCOD/L concentrations, with the aim of assessing the influence of the lactic acid source, recycled from PLAc or synthetically sourced, on chain elongation performance. A 3 M NaOH solution was added as needed to neutralize lactic acid addition and maintain the pH at an optimal level of 5.5 ± 0.2 to start the chain elongation tests. The reactors were closed and flushed with N_2_ to allow chain elongation in an anaerobic environment. Daily (except on weekends), around 2 mL of broth was withdrawn with a syringe to measure pH, carboxylic acid/ethanol content after filtration through nylon syringe filters (0.22 μm porosity), and total lactate consumption after filtration through nylon syringe filters (0.45 μm porosity).

### 2.6 Chemical Characterization

Bioplastics were dried in an oven at 45 °C before the chemical characterization.

The Total and Volatile Solids (TS and VS) content, the Total Chemical Oxygen Demand (COD_t_) of the bioplastic samples, and the total carbohydrates content (Carbs_t_) of the SBB were measured as described in Angelini et al. (2025).^25^ SBB was also dissolved at temperatures ranging from 40 to 70 °C, keeping the solutions under stirring for 45 min, 60 min, and 120 min with the aim of evaluating the effect of time to temperature ratio on the detection of Carbs_t_. On the other hand, COD_t_ of PLAc was measured by dissolving around 5 mg of the sample in the COD Cell Test 500–10000 mg/L (Spectroquant Merck) and adding the proper amount of water according to the kit instructions.

Soluble COD (COD_s_) of hydrolysates from SBB and PLAc HTP was also analyzed through the COD Cell Test (Spectroquant Merck). Soluble carbohydrate content of SBB hydrolysate after HTP and fermentative broths after 8 days from substrate feeding was measured as described in Gallipoli et al. (2020).^48^ Carboxylic acid/ethanol content of supernatants after biomass washing cycles, broths during acidogenic fermentation, and chain elongation tests (0.22 μm porosity-filtered fractions) were analyzed through an Agilent 8860 Gas-Chromatograph equipped with a flame ionization detector (FID), as described in Montecchio et al. (2024).^39^ Total lactate was determined in the 0.45 μm-filtered soluble phase of broths during chain elongation tests using the d/l-lactic acid assay kit.

Calculations:

Yield of carbohydrates extracted from SBB (*Y*_SBB_) and lactic acid from PLAc (*Y*_PLAc_) was calculated as follows (eq 1 and eq 2, respectively):

1 $$Y_{\mathrm{SBB}}=\mathrm{Extracted Carbohydrates}/\mathrm{Total Carbohydrates}\times 100$$

2 $$Y_{\mathrm{PLAc}}=\mathrm{Extracted Lactic Acid}/\mathrm{Total Lactic Acid}\times 100$$

Concentration of each metal (Concentration of metals) present in PLAc hydrolysate was calculated as described below (eq 3):

3 $$\mathrm{Concentration of metals}=\left(\mathrm{Metal}\times V_{e}\right){/m}_{e}$$

where *V*_e_ is the volume of hydrolysate, and *m*_e_ is the mass of hydrolyzed PLA (calculated as the complement to that of the initial mass and the residual mass of PLAc after HTP).

Product selectivity of fatty acids (FAs), (Product selectivity (*t*_n_)), evaluated at a specific time during acidogenic fermentation, was calculated as described in eq 4:

4 $$\mathrm{Product selectivity}\;\left(t_{n}\right)=\mathrm{FA}/\mathrm{Total FAs}\times 100$$

Yield of SCFAs (*Y*_SCFAs_) achieved after anaerobic fermentation was calculated as described in eq 5:

5 $$Y_{\mathrm{SCFAs}(C2-C5)}=\overset{i=C5}{\underset{i=C2}{\sum }}\left(C_{i,t8}\times V_{t8}-C_{i,t0}\times V_{t0}\right)/{\mathrm{COD}}_{\mathrm{fed}}$$

where *C*_*i*_,_*t*8_ is the concentration of carboxylic acid (*C*_*i*_ = C2–C5) produced after 8 days from feeding, and *C*_*i*_,_*t*0_ is the concentration of residual carboxylic acid after PBS cleansing of biomass at the start of the test; *V*_*t*8_ and *V*_*t*0_ are the volumes of the reactor at day 8 and 0, respectively; COD_fed_ is the amount of substrates, COD basis, fed at the start of the test, including glucose or SBB-based carbohydrates from HTP.

Yield of caproic acid (*Y*_C6_) achieved after chain elongation was calculated as described in eq 6:

6 $$Y_{C6}=\left(C_{6,\mathrm{tf}}\times V_{\mathrm{tf}}-C_{6,t0}\times V_{t0}\right){/\mathrm{COD}}_{\mathrm{fed}}$$

where *C*_6_,_*tf*_ is the concentration of caproic acid produced at the end of CE, and *C*_6_,_*t*0_ is the concentration of caproic acid when lactic acid was fed; *V*_*tf*_ and *V*_*t*0_ are the volumes of the reactor at the end and start of the CE test; COD_fed_ is the amount of lactic acid, COD basis, fed at the start of the test, including synthetic and PLAc-recycled lactic acid.

Conversion of the carbon entering the bioprocess into MCFAs (*C*_conv,MCFAs_) was calculated as in eq 7

7 $$C_{\mathrm{conv},\mathrm{MCFAs}}{=\mathrm{COD}}_{\mathrm{out}}\left(\mathrm{MCFAs}\right)/\left({\mathrm{COD}}_{\mathrm{in}}\left(\mathrm{SBB}\right)+{\mathrm{COD}}_{\mathrm{in}}\left(\mathrm{PLAc}\right)\right)$$

## 3 Results and Discussion

### 3.1 Bioplastics Chemical Characterization

In order to verify whether the selected bioplastic waste was suitable for recycling and upcycling routes, a basic characterization was performed, including the determination of parameters such as TS, VS, Carbs_t_, and COD_t_. The characterization of postconsumer SBB and PLAc samples, therefore, was carried out, and the obtained results are presented in Table 1.

**Table 1:** Characterization of SBB and PLAc

	SBB	PLAc
Thickness, μm	17 ± 3	21 ± 4
TS, g/kg	994	999
VS, g/kg	989	998
VS/TS, %	99.4	99.8
Carbs_t_, gCOD/kg	200 ± 20	*n.a.[Table-fn tbl1fn1]*
COD_t_, gCOD/kg	2450 ± 200	1100 ± 100

^a^ *n.a.* not analyzed.

Regarding TS, no significant differences were detected between the two bioplastics. Following the drying at 45 °C, the moisture content remained below 7 g/kg, although slightly higher values were measured for SBB. This modest difference is likely due to the more pronounced hygroscopic nature of the starch-based material compared to PLAc. Both samples exhibited a VS/TS ratio exceeding 99%, confirming that the materials are composed almost entirely of organic matter, with only a negligible amount of inorganic constituents, such as mineral fillers or metal-based functional additives typically incorporated during bioplastic formulation and processing. While most studies reported in the literature focus on the laboratory-produced blends containing TPS ranging from 20 to 80 wt %,^49,50^ information regarding the composition of commercially available starch-based products remains limited. The content of total carbohydrates of the postconsumer SBB used in this study amounted to around 200 gCOD/kg, corresponding to slightly less than 20 wt %, confirming what was reported in Angelini et al. (2025).^25^ Optimization of the carbohydrate analysis revealed that prolonged acid-catalyzed dissolution of SBB negatively impacts yield. Results showed that carbohydrate detection remained consistent for up to 45 min; however, extending the process to 60 or 120 min, particularly within the 40–70 °C range, led to measurable degradation of the starchy fraction. These findings suggest that to prevent underestimation of the carbohydrate content, the dissolution step must be restricted to 45 min, as exceeding these parameters triggers the degradation of sensitive organic constituents. The total COD content measured in SBB (2450 gCOD/kg) was more than twice that measured in PLAc (1100 gCOD/kg), suggesting a significantly higher density of oxidizable organic carbon in the starch-based blend. Such a property increases its potential for upcycling the carbon into carboxylic acids both from a stoichiometric and a kinetic perspective. In fact, SBB contains thermoplastic starch with a modified structure in addition to the blending with other polymers (PBAT, PCL, or PLA), which results in a more amorphous matrix compared to the semicrystalline hydrophobic PLAc, which undergoes slower hydrolysis.^51,52^

### 3.2 Bioplastics Chemical Recycling: Hydrothermal Pretreatment

The aspect of SBB scraps before and after HTP at 134 °C for 20 min is reported in (Figure S1 Supporting Information).

After HTP, the SBB size appeared slightly reduced, and the distinguishable square-shaped scraps were no longer evident because of high conglomeration. In addition, no evident color changes of SBB scraps were observed. SBB retained its original green color. In Table 2, the yield of carbohydrates extracted from SBB (*Y*_SBB_), varying the concentration from 1w/v%, 2w/v%, and 3w/v% and temperature/duration conditions, was reported.

**Table 2:** Yield of Carbohydrates Recovered from SBB through HTP (*Y*_SBB_ in wt %)

	1 w/v%	2 w/v%	3 w/v%
98 °C, 20 min	45 ± 0.7	39 ± 7.1	50 ± 0.8
98 °C, 60 min	56 ± 2.3	66 ± 0.9	58 ± 1.2
134 °C, 20 min	86 ± 8.1	6 5 ±0.7	33 ± 1.4

The carbohydrate extraction yields reported in Table 2 demonstrate a nonlinear relationship among temperature, residence time, and solid loading (w/v%), which is characteristic of the complex hydrolysis behavior of bioplastics. At the moderate temperature of 98 °C, a residence time of only 20 min was sufficient to achieve an average carbohydrate extraction efficiency (*Y*_SBB_) of 45 ± 6 wt % across the different bioplastic-to-water concentrations. Increasing the residence time from 20 to 60 min led to a consistent improvement in yield at all tested concentrations, with the 2% w/v condition showing the largest improvement (from 39 ± 7.1% to 66 ± 0.9%). This suggests that at sub-boiling temperatures, the process is kinetically limited, requiring longer durations to overcome the structural integrity of the thermoplastic starch matrix.

In contrast to residence time, which significantly affected extraction efficiency, the influence of bioplastic concentration on *Y*_SBB_ was negligible at 98 °C. A pivotal shift in behavior was observed at 134 °C. While the 1% w/v loading achieved the highest extraction yield efficiency (86 ± 8.1%), yield decreased sharply with increasing solid concentration, reaching only 33 ± 1.4% at 3% w/v. This inverse correlation at elevated temperatures may be attributed to mass transfer limitations and/or the degradation of the released sugars. At 134 °C, in fact, the rapid structural transformation of the SBB material at 3% w/v likely created a dense, “shielded” polymer mass that prevented the water from penetrating the inner regions of the particles, thereby limiting hydrolysis and reducing carbohydrate recovery. Moreover, higher bioplastic concentrations at high temperatures may lead to sugar saturation and potential thermal transformation, contributing to a significantly lower measured yield.

A disintegration degree of around 25 ± 5% was recorded among the tests, and the maximum value calculated was attributed to the thermally hydrolyzed SBB at 134 °C for 20 min at a concentration of 1% w/v. This demonstrates that the latter was the most affected by the treatment and that the higher temperature favored depolymerization and solubilization of bioplastic.^53^

The alkaline HTP of rigid PLAc at 134 °C for 180 min resulted in a profound acidification of the reaction medium, with the pH shifting from an initial 11.5 (±0.5) to a final 2.9 (±0.2), providing evidence that lactic acid extraction took place. The mild conditions of temperature and pressure regimes yielded more modest PLAc hydrolysis efficiencies with yields not exceeding 40% regardless of the solid-to-liquid ratio (w/v%). These findings align with current literature; for instance, Strik and Heusschen (2023)^17^ reported an average PLA hydrolysis efficiency of approximately 38%. However, it is noteworthy that their results were achieved at a significantly lower temperature (70 °C) but required an extended residence time of 21 days.

*Y*_PLAc_ values decreased with increasing PLAc concentrations, while the residual mass increased, in particular at 1.5 w/v% concentration. This result is in contrast with what was reported by Strik and Heusschen (2023)^17^ who achieved the highest volumetric hydrolysis rate at the highest amount of PLA loaded. Discrepancy likely arises from differences in the experimental setup or the specific surface-to-volume ratio of the PLAc scraps used. However, the results of the present study align closely with Yagihashi et al. (2010),^54^ who reported that increasing PLA concentrations, alkaline hydrolysis efficiency is significantly inhibited. They attributed this phenomenon to the fact that the reaction proceeds via a surface-erosion mechanism, where hydroxyl ions attack the polymer chains at the solid–liquid interface. This surface-centric pathway has been corroborated by a recent study,^55^ confirming that for rigid PLA applications like tableware, the accessible surface area, and not the total mass, is the limiting factor for lactic acid recovery. At higher concentrations (e.g., 1.5 w/v%), the available surface area relative to the total liquid volume may be insufficient to maintain high conversion rates, leading to yield plateaus and increased residual mass. The highest disintegration degree of 40 ± 5%, in fact, was recorded for hydrolyzed PLAc at 0.5 and 1 w/v% concentrations and decreased to 30 ± 2% at 1.5 w/v%. These results demonstrate that PLAc hydrolysis is induced by high water content, which promotes the cleavage of ester bonds and further proceeds via an autocatalytic mechanism driven by the terminal carboxyl groups. The concentration of metals in the PLAc hydrolysate in μg/g of extracted lactic acid is reported in (Table S1 Supporting Information).^56^

The primary source of metals in plastics has been attributed to contamination from additives, stabilizers, catalysts, and coatings.^57^ Metal-based additives may be insoluble inorganic particles, incorporated as fillers, or partially/totally soluble organometallic compounds. In this study, the PLAc hydrolysate revealed a complex metallic profile: while Si, Na, Ca, B, Al, Mg, and K were consistently detected above the method detection limit, transition metals and alkaline earth metals such as Ba, Cu, Fe, Mn, Sr, and Zn occurred at lower frequencies. The prevalence of Si, Ca, Al, and Mg is particularly noteworthy, as these elements are typically employed as inert fillers and stabilizers in polymer formulations.^58^ The detection of these metals suggests that the hydrolytic breakdown of the PLAc matrix facilitates the release of the formerly encapsulated inorganic constituents. Calcium carbonate is, for instance, a common mineral filler used to increase the stiffness of plastic-based materials, while boron nitride is often used to improve the mechanical properties of PLA-based composites.^59^ The integration of inorganic fillers, specifically metal oxides such as ZnO, Al_2_O_3_, MgO, and SiO_2_, is a well-established manufacturing practice to boost the thermomechanical properties of neat PLA. In particular, the reinforcement provided by SiO_2_ was found to be a useful filler for improving the mechanical performance of polymeric materials.^60^ Beyond structural reinforcement, the elemental composition of the medium plays a pivotal role in modulating microbial metabolic pathways. The literature suggests that certain metallic species, such as iron (Fe) and potassium (K), can actually serve as metabolic catalysts to facilitate acidogenic fermentation by promoting extracellular electron transfer, thereby accelerating the breakdown of complex organic matter.^61,62^ However, the metabolic efficacy of these microbial consortia is inherently constrained by rigorous inhibition thresholds. As Ji et al. (2022)^63^ observed that high levels of Na^+^ (12.5 g/L) exerted inhibitory effects on chain elongation, while K^+^ ions are generally well-tolerated. Crucially, the Na^+^ levels detected in this investigation remained substantially below the critical threshold. The impact of metal ions released during PLAc hydrolysis on microbial kinetics may lay the groundwork for future studies and will be the subject of further investigation.

The following paragraphs evaluate the valorization of extracted carbohydrates from the HTP of SBB for the production of SCFAs. These intermediates were subsequently elongated to MCFAs via chain elongation by using lactate extracted from PLAc HTP as an electron donor.

### 3.3 Recycling of Extracted SBB Carbohydrates for SCFA Production

The efficiency of carboxylic acid removal from biomass through PBS cleansing cycles is reported in (Figure S2 Supporting Information).

Following dispersal in a basal medium and a subsequent acclimation phase, the cleansed microbial biomass was supplemented with 386 ± 14 mg of glucose (Control reactor) and the same amount of carbohydrates recovered from the HTP of SBB, reaching a starting carbon concentration of 855 ± 44 mgCOD/L in the reactors. The resulting production trends for carboxylic acids and ethanol (EtOH) are depicted in Figure 3a (Control reactor) and Figure 3b (Test reactor). The baseline metabolic activity of the Blank reactor (without feed) is documented in (Figure S3 Supporting Information).

**Figure 3:**
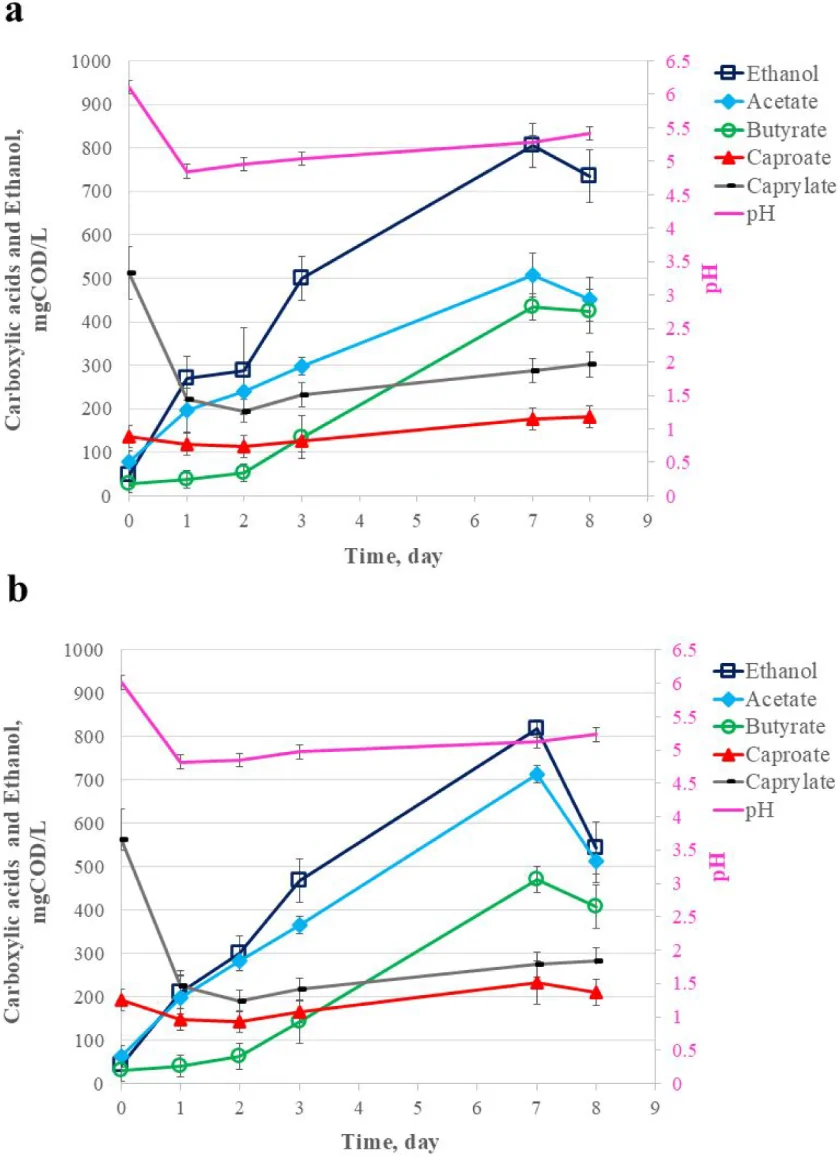
Carboxylic acids/ethanol content and pH trend in a) Control and b) Test reactors during the acidogenic fermentation test (ethanol: blue square; acetate: light blue rhombus; butyrate: green circle; caproate: red triangle; caprylate: dark gray dash; pH: pink solid line). Error bars represent the standard deviation.

Within 24 h of substrate supplementation, a sharp decline in pH, from 6.0 to below 5.0, was observed across all experimental groups. Notably, this acidification trend was independent of the carbon source, with both synthetic glucose and SBB-extracted carbohydrates yielding nearly identical profiles. Following this initial drop, the pH stabilized slightly above 5.0. This rapid acidification is characteristic of the onset of the acidogenic fermentation process, as indicated by the production of acetate, which reached approximately 200 mgCOD/L within the first day. Both the glucose and the extracted SBB carbohydrates were effectively assimilated by the microbial inoculum, resulting in the conversion to ethanol and SCFAs within a seven-day period. The remaining substrates were around 23 ± 0.5 mgCOD/L and 30 ± 0.9 mgCOD/L in the Control and Test reactors, respectively. The *Y*_SCFAs_ values achieved in Control and Test reactors were 730 ± 15 mgCOD/gCOD_fed_ and 714 ± 20 mgCOD/gCOD_fed_, respectively. Interestingly, the microbial inoculum consumed glucose and SBB-based carbohydrates achieving statistically indistinguishable SCFA yields, similar to the one reported by Kumar and Venkata Mohan (2018)^64^ who reached around 644 mgCOD_SCFAs_/gCOD_fed_ from fermentation of reducing sugars extracted from thermochemically pretreated vegetable waste. The use of a mixed inoculum likely promoted a wide range of microbial metabolic transformations. The fermentation findings showed that up to 4 different SCFAs were produced (acetate, propionate, isobutyrate, butyrate). Ethanol, acetate, and butyrate were the main products found. Minor constituents were excluded from Figure 3 to maintain clarity in the visualization of dominant carbon flows. In the early fermentation stage, ethanol and acetate predominated, likely due to the acetate–ethanol-type fermentation pathway, which commonly occurs when readily organic substances are available. Ethanol was produced at a higher production rate and steadily accumulated up to approximately 800 mgCOD/L, contrary to what was reported by Zeng et al. (2025),^24^ who showed a rapid consumption of ethanol after 2 days of fermentation in favor of butyrate production. This discrepancy likely highlights a concentration threshold required to trigger the chain elongation bioprocess. In the study by Zeng et al. (2025),^24^ ethanol concentration was very high (∼3000 mgCOD/L) due to the use of concentrated hydrolysates, which probably provided the necessary thermodynamic driving force for its use as an electron donor. Besides, in the pH range from 5.0 to 5.5, formation of acetate was favored, reaching a peak accumulation of 500 mgCOD/L within 8 days (Figure 3b). Following a 2-day lag phase, the metabolic profile shifted toward mixed-acid fermentation, characterized by the rapid production of butyrate up to around 400 mgCOD/L over the following 5 days. The analysis of the product selectivity further elucidated this transition. Acetate remained the primary carboxylic acid, keeping a stable product selectivity of 45% between days 3 and 7. On the other hand, butyrate selectivity increased from 10% on day 3 to 22% on day 7. This shift indicates a transition toward butyric acid fermentation as the dominant metabolic process. This pathway was particularly active as the system stabilized at pH 4.8–5.0, aligning closely with the optimal pH range for butyric acid fermentation reported by Feng et al. (2020).^65^ Biohydrogen-specific production after 8 days of acidogenic fermentation was 66 ± 4 and 61 ± 3 mL H_2_/g VS_carb_ for Test and Control reactors, respectively, typical for sugar fermentation at pH < 5.^64^

The initial presence of long-chain carboxylic acids, specifically caproates and caprylates, was attributed to residual concentrations from the initial inoculum. Despite the preliminary accurate washing steps with PBS, the hydrophobic nature of these compounds likely hindered their complete removal.

Caproate concentration remained stable at around 170 mgCOD/L without any significant fluctuations over the entire duration of the test. Caprylate was initially present at higher concentrations; however, it declined sharply within the first day and subsequently leveled off at around 270 mgCOD/L until the end of the test.

### 3.4 Recycling of Extracted PLAc Lactate to Produce MCFAs through Chain Elongation

The mixed-culture biomass effectively converted both synthetic and recycled lactic acid into a range of carboxylates (Figure S4, Supporting Information; Figure 4a–d). For better clarity, the diagram of potential metabolic pathways involved in substrate utilization and conversion is presented in Figure S5 in the Supporting Information.

**Figure 4:**
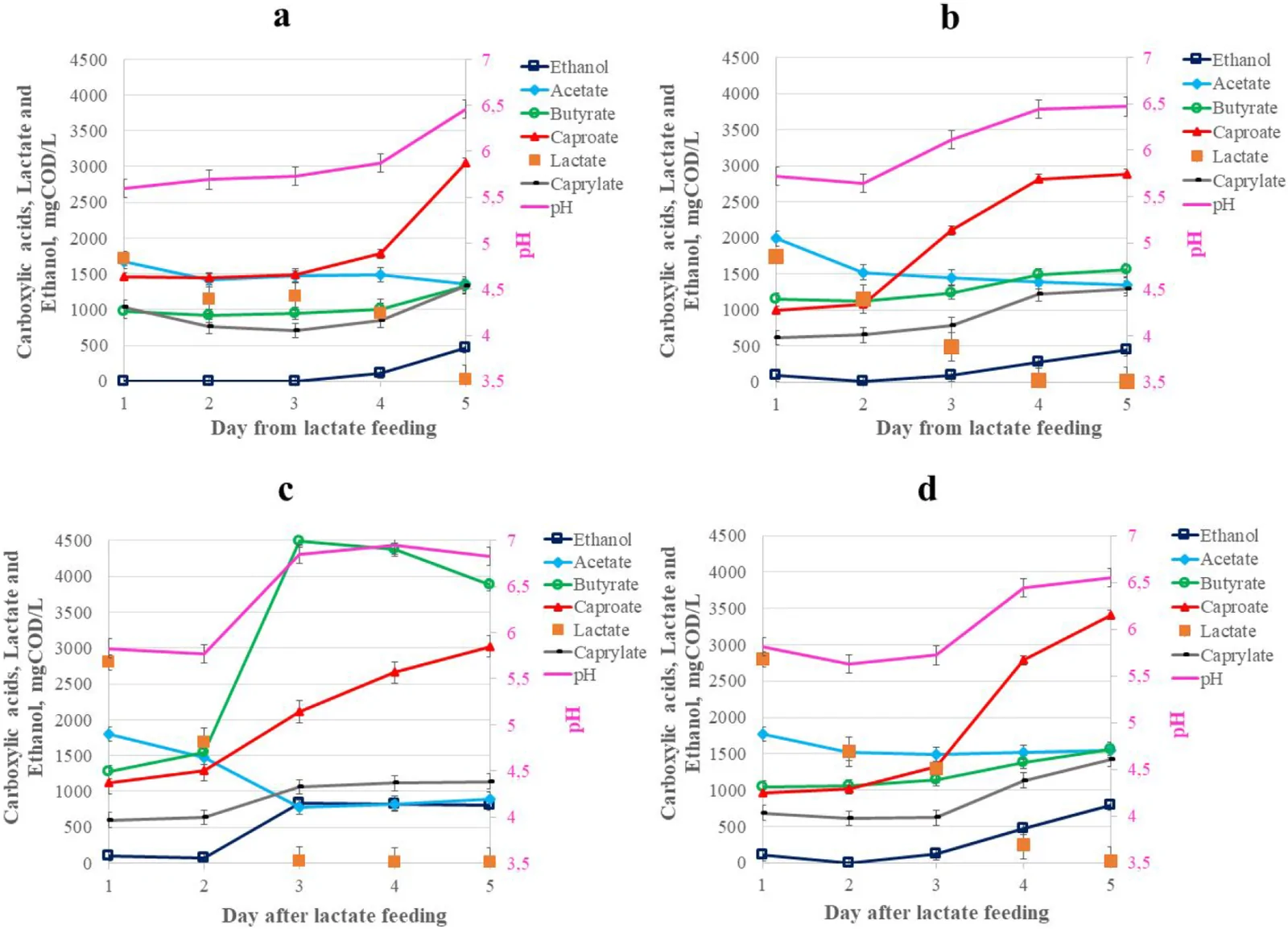
Carboxylic acids/ethanol and pH trend in a) and c) Control reactors starting respectively with 1738 ± 25 mgCOD/L and 2780 ± 18 mgCOD/L synthetic lactic acid; and b) and d) Test reactors starting respectively with 1717 ± 30 mgCOD/L and 2798 ± 25 mgCOD/L recycled lactate during the chain elongation test (ethanol: blue square; acetate: light blue rhombus; butyrate: green circle; caproate: red triangle; lactate: orange dots; caprylate: dark gray dash; pH: pink solid line). Error bars represent the standard deviation.

On day 8 after the acidogenic fermentation tests, synthetic lactic acid was fed (Figure S4a, b). It is worth underlining that the absence of lactic acid in the reactors was verified before the start of the feeding. After 2 days from lactic acid feeding, about 50% of it was converted to acetate, butyrate, and caproate, with a caproate product selectivity of 29% and 20% in the Control and Test reactors, respectively. The co-occurrence of acetate production may be attributed to the oxidation of lactic acid and/or the simultaneous reduction of the CO_2_ developed through the Wood–Ljungdahl pathway.^66^ After a deliberate long interval, the experimental lactic acid feeding, focus of this investigation, was done (Figure 4a, b, c, d).

In the first stage, pH increased gradually due to a slow and partial consumption of lactic acid (Figure 4a), completely depleted at the end of the test, mirrored by the increase of pH up to 6.5. Lactic acid was, in fact, used as an electron donor for the chain elongation of acetate into butyrate and then into MCFAs, such as caproate and caprylate. The curve slope of caproate (Figure 4a) is almost zero at first, then becomes moderately positive, and finally turns very steep on the last day, indicating a sudden and accelerated increase in caproate concentration. Caproate displayed in Figure 4b increases more gradually with a slope that remains relatively uniform over time, analogous to the pH trend. In both reactors, caproate reached a peak concentration of approximately 3000 mgCOD/L with a product selectivity of around 35%, complemented by a moderate accumulation of caprylate, which peaked at roughly 1300 mgCOD/L. The calculated product yield for caproate was 0.87 and 1.05 gC6/glactate_fed_ (on COD basis) for Control and Test reactors, respectively. The ability of the system to sustain MCFAs production upon addition of PLAc-derived lactate further supports the functional presence of lactate-utilizing chain-elongating microorganisms. The absence of detectable delays, acid accumulation, or productivity collapse following the addition of recycled hydrolysates suggests that, under the tested conditions and concentrations, no acute inhibition occurred that prevented chain elongation.

Similar metabolic trends were observed in Figure 4c and 4d, where a feedstock of approximately 2780 mgCOD/L of lactic acid successfully promoted the production of MCFAs, with a similar final caproate concentration to the one achieved at low lactic acid load (Figure 4a and 4b). However, the calculated yields were 0.65 and 0.84 gC6/glactate_fed_ (on COD basis) for Control and Test reactors, respectively, significantly lower with respect to the ones obtained at lower lactate loading. Furthermore, the excess lactic acid provided in the higher-load trials (Figure 4c, d) appeared to be channeled also toward caprylate synthesis (1300 mgCOD/L) or maintained as residual substrate, indicating a metabolic ceiling on caproate accumulation under the tested pH conditions. This suggests that caproate production in these systems was governed by thermodynamic limitations or product inhibition rather than substrate availability. Strik and Heusschen (2023)^17^ also reported the production of carboxylates by adding recycled lactic acid (up to 15.5 g/L) derived from PLA food packaging. However, in that study, butyrate was the dominant product (reaching up to 6.5 g/L), while medium-chain carboxylates like caproate were produced in much lower, nondominant quantities. This predominance of butyrate may be attributed to the absence of SCFAs in the fermentative broth once lactic acid was fed. In contrast, in the present study, SCFAs are produced and are already available for chain elongation. This highlights the importance of the ratio between the electron donor and SCFAs, particularly the lactate to acetate ratio, which in this study is equal to 1:1 (mM C basis). Acetate produced and accumulated plays an important role in chain elongation, as shown in the study by Brodowski et al. (2022),^67^ where a predominance of butyrate and caproate was strictly related to the lactate to acetate ratio and was higher when it was equal to 1:1 (mM C basis). Similarly, the lactate to butyrate ratio influences caproate accumulation, as Nzeteu et al. (2022).^46^ In view of system’s upgrading, further feeding with acidic PLAc hydrolysate could be used to control the pH raised to almost 7 during chain elongation, by reducing the need for chemicals for buffering and boosting the production of additional valuable carboxylates.

The key advance of the present work is the demonstration of a new system-level valorization strategy enabled by decoupling chemical deconstruction from microbial synthesis while maintaining functional integration. While previous integrated or semi-integrated systems largely stop at acetate and butyrate, this study demonstrates the feasibility of pushing carbon flux toward higher-value MCFAs (caproate and caprylate) directly from postconsumer bioplastics, highlighting robustness toward real-product complexity, an aspect rarely addressed in earlier studies.

On the basis of the available characterization in terms of COD content (Table 1) and the entering and exiting streams from each process unit, a simplified carbon balance was drafted to gain a better understanding of how carbon was used and converted by the different processes (Table 3). On the basis of the experimental results, the integrated process enables the recovery and upgrading of a significant fraction of the carbon originally embedded in the bioplastic materials. In fact, hydrothermal treatment extracted 10% of the available carbon (as sugars), which was subsequently fermented into SCFAs and ethanol, providing the carbon backbone for chain elongation, and 12% of the polymer carbon as lactic acid from PLAc. During the anaerobic cascade fermentation and chain elongation, carbon was redistributed primarily to caproate and caprylate. When expressed on a COD basis, the combined process converts 78% of the total carbon entering the bioprocess into MCFAs, with the remainder mainly as residual SCFAs and biomass.

**Table 3:** Carbon Balance of the Integrated Process by Recycling Mixed SBB and PLAc Waste

	Amount (kg)	COD_t_ (g)	COD_in_ ^a^ (g)	COD_out_ (SCFAs) (g)	COD_out_ (MCFAs) (g)	*C*_conv, MCFAS_ (%)
SBB	1	2450	255	182		78
PLAc	1	1100	134		168	

a Corresponding to COD_s_ of hydrolysates from SBB and PLAc.

Overall, this study provides a proof-of-concept demonstration of an integrated chemo-microbial route for the upcycling of real bioplastic waste into MCFAs. While the results highlight the technical feasibility and robustness of the approach, several challenges remain to be addressed for future scale-up and practical implementation. In particular, the hydrothermal and alkaline pretreatment steps, although effective, require careful optimization to reduce energy demand and improve process efficiency. Mass transfer limitations observed at higher solid loadings and the partial hydrolysis efficiency of PLA indicate the need for improved reactor design and operating conditions. Furthermore, the stability and control of the biological stages, including pH regulation, substrate balance, and potential inhibition from released additives or inorganic constituents, represent critical aspects for continuous operation. Process integration also poses additional challenges, such as the development of fed-batch or continuous configurations. Indeed, a fed-batch system alternating feeding cycles of recycled carbohydrates from TPS-based blends and recycled lactic acid from PLA could be explored in future studies, including biomass retention via granular sludge or biofilm systems. Addressing these bottlenecks will be essential to enhance process scalability and economic viability. Nevertheless, the present work lays a solid foundation for the development of circular biorefinery concepts based on heterogeneous urban bioplastic waste streams, supporting future advancements toward industrial application.

## 4 Conclusions

This study demonstrated a proof of concept for an alternative end-of-life strategy for real bioplastic waste, thermoplastic starch-based bags and polylactic acid cups, using a combined chemo-microbial recycling approach. Thermal hydrolysis at 134 °C for 20 min of bags enabled the extraction of more than 80% of the carbohydrates, while alkaline hydrolysis of cups yielded approximately 40% of the initial lactic acid under the same temperature and extended reaction time. The carbohydrates extracted from the bags were rapidly and efficiently converted into short-chain fatty acids, mainly acetate and butyrate, via mixed-acid fermentation. In a sequential chain-elongation process, lactic acid recovered from polylactic acid–based cups was oxidized, leading to the conversion of short-chain fatty acids into approximately 3000 mgCOD/L of caproate and 1300 mgCOD/L of caprylate.

This study provides valuable insights into the valorization of bioplastic waste as feedstock for the production of C2–C4 carboxylates, which may undergo chain elongation toward valuable C6–C8 products (approximately 85 g MCFAs (COD) per kg of mixed bioplastics). As this study represents a proof of concept, several technical and scientific challenges remain to be addressed before industrial implementation can be fully assessed. In particular, further optimization of the PLA hydrothermal pretreatment is required to maximize lactate recovery. A deeper characterization of compounds during bioplastics hydrolysis is also needed, as potential byproducts may exert inhibitory effects on microbial activity and affect product selectivity. From a scale-up perspective, additional efforts should focus on managing the heterogeneity of postconsumer bioplastic waste streams and ensuring the stability and robustness of the coupled fermentation and chain elongation processes under continuous operation. Despite these challenges, the results demonstrate the feasibility of an integrated two-stage biotechnological recycling approach in which the carbon recovered from bioplastic waste is converted into valuable platform chemicals. By reducing resource losses and promoting the valorization of urban bioplastic waste streams forecasted to increase over the next years, this strategy may contribute to the transition toward a more circular bioeconomy.

## Supplementary Material



## Notes

Macroscopic appearance of SBB scraps before and after HTP at 134 °C, 20 min (Figure S1), concentration of metals in PLAc hydrolysate (Table S1), soluble carboxylic acids content in pristine biomass and in supernatants after washing cycles with PBS; error bars represent the standard deviation (Figure S2), carboxylic acids/ethanol content and pH trend in Blank reactor during the acidogenic fermentation test (ethanol: blue square; acetate: light blue rhombus; butyrate: green circle; caproate: red triangle; caprylate: dark gray dash; pH: pink solid line); error bars represent the standard deviation (Figure S3), carboxylic acids production in a) Control and b) Test reactors starting with 1070 ± 4.1 mgCOD/L synthetic lactic acid (ethanol: blue square; acetate: light blue rhombus; butyrate: green circle; caproate: red triangle; lactate: orange dots; caprylate: dark gray dash; pH: pink solid line); error bars represent the standard deviation (Figure S4), diagram of metabolic pathways for bioplastics upcycling through acidogenic fermentation and chain elongation (Figure S5) (PDF)
