# Supplementary material for: Green Route for the Upcycling of Urban Bioplastic Waste: A Biotechnological Resource Recovery Strategy toward Medium-Chain Fatty Acids Production
Source: ACS Environ Au. 2026 Jul 22;6(5):879–89. doi: 10.1021/acsenvironau.6c00074 (PMC13602330; doi:10.1021/acsenvironau.6c00074)
Supplement: Supplementary file 1 [file vg6c00074_si_001.pdf]

Supporting information

Green route for the upcycling of urban bioplastic waste: a biotechnological resource recovery strategy towards medium-chain fatty acids production

Stefania Angelini[1]*, Agata Gallipoli[1], Andrea Gianico[1], Francesca Angelini[1], Michela Sbicego[1,2], Vincenzo Piemonte[3], Camilla M. Braguglia[1]

[1]Water Research Institute, National Research Council (CNR-IRSA), Strada Provinciale 35d, 9, 00010, Montelibretti (RM), Italy

[2]Department of Chemical Engineering, Materials, Environment, Sapienza - University of Rome, Piazzale Aldo Moro 5, 00185, Rome, Italy

[3]University Campus Bio-Medico of Rome, Via Alvaro del Portillo 21, 00128, Rome, Italy

*Corresponding author: Stefania Angelini
e-mail: stefania.angelini@irsa.cnr.it
phone: +39 0690672355

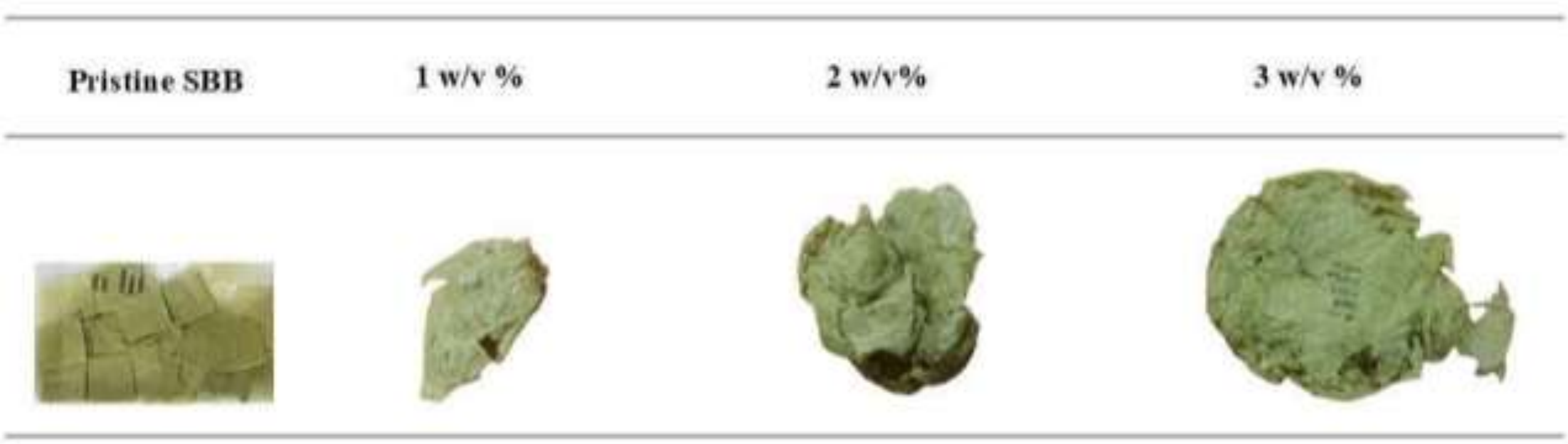

Figure S1. Macroscopic appearance of SBB scraps before and after HTP at 134 °C, 20 min.

Table S1. Concentration of metals in PLAc hydrolysate.

Type of metal	Concentration of metals, µg/g
Al	0.42
B	1.39
Ba	0.009
Ca	1.85
Cr	0.0002
Cu	0.0032
Fe	0.0166
Hg	0.0003
K	0.37
Mg	0.44
Mn	0.0027
Na	53.4
Ni	0.00002
Si	125.3
Sr	0.02
Zn	0.00379

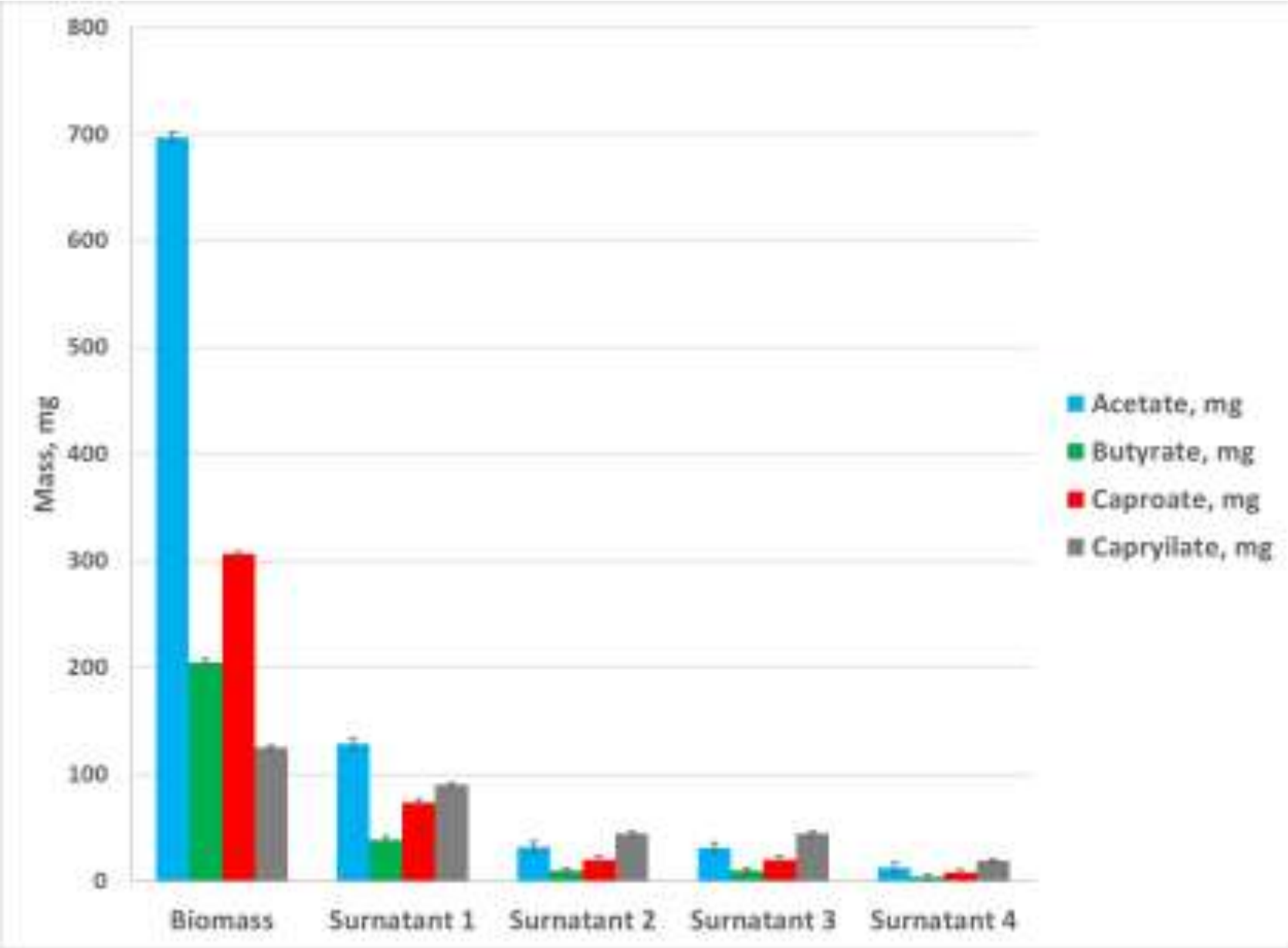

Figure S2. Soluble carboxylic acids content in pristine biomass and in surnatants after washing cycles with PBS. Error bars represent the standard deviation.

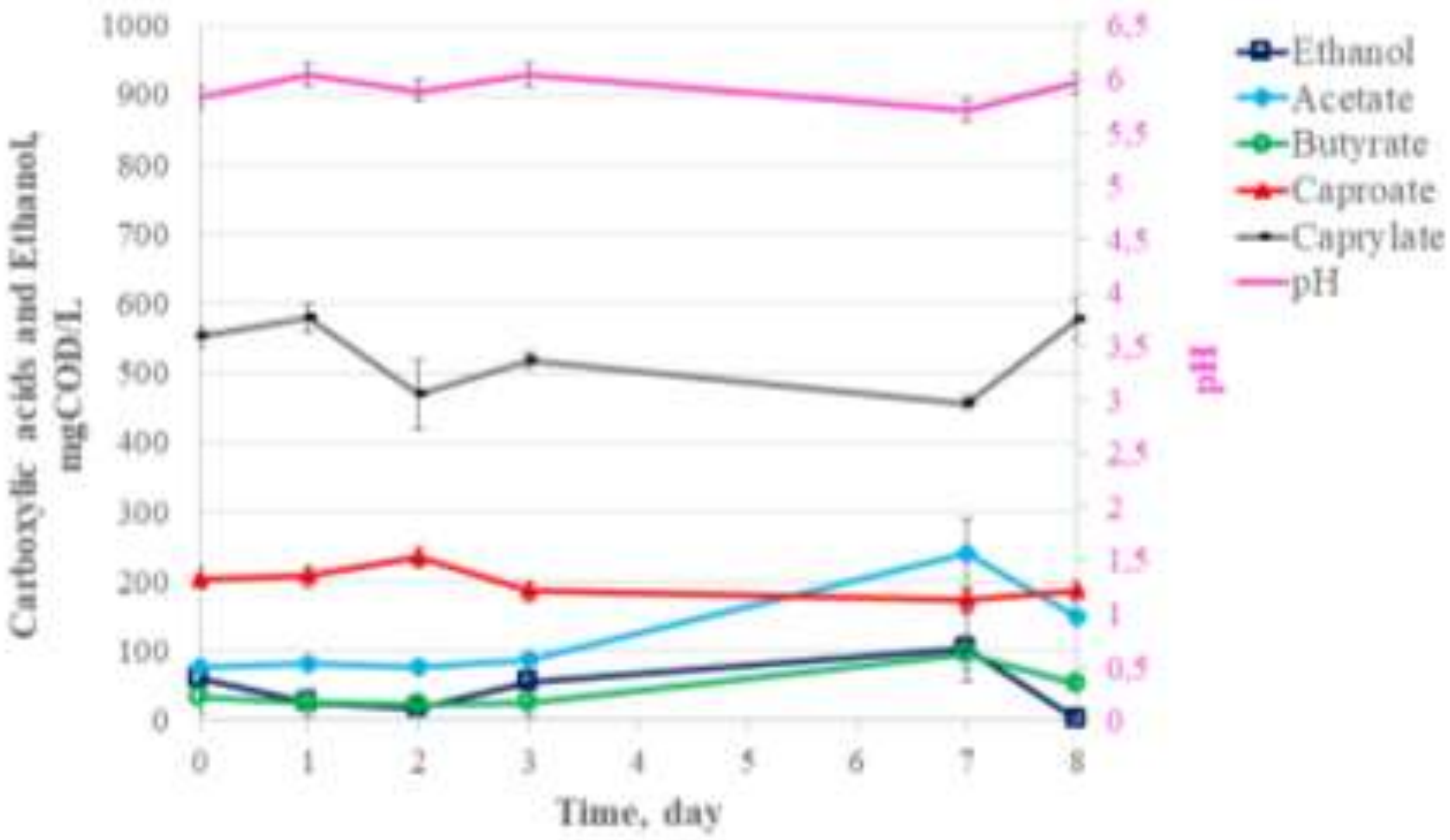

Figure S3. Carboxylic acids/ethanol content and pH trend in Blank reactor during the acidogenic fermentation test (ethanol blue square; acetate light blue rhombus; butyrate green circle; caproate red triangle; caprylate dark grey dash; pH pink solid line). Error bars represent the standard deviation.

a

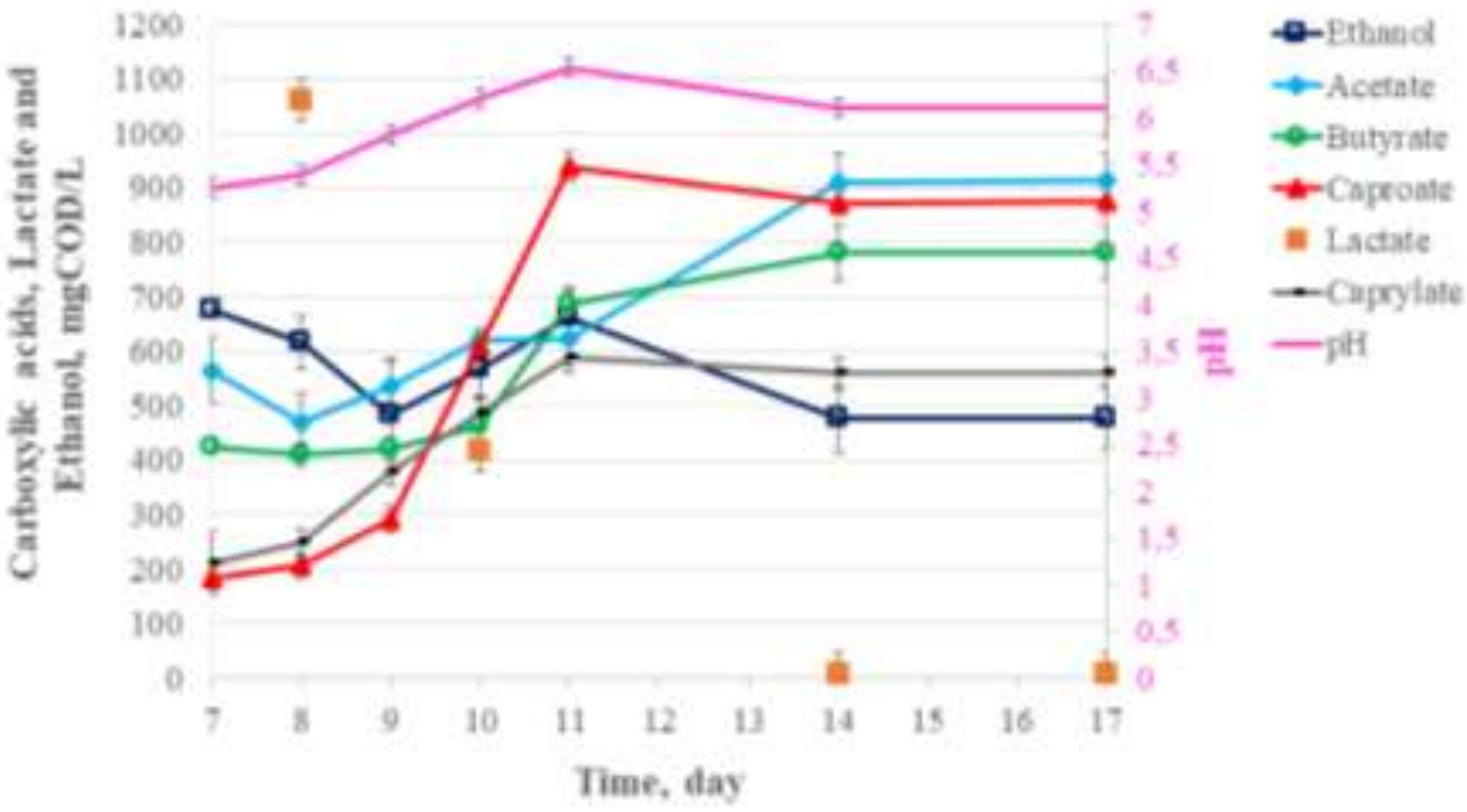

b

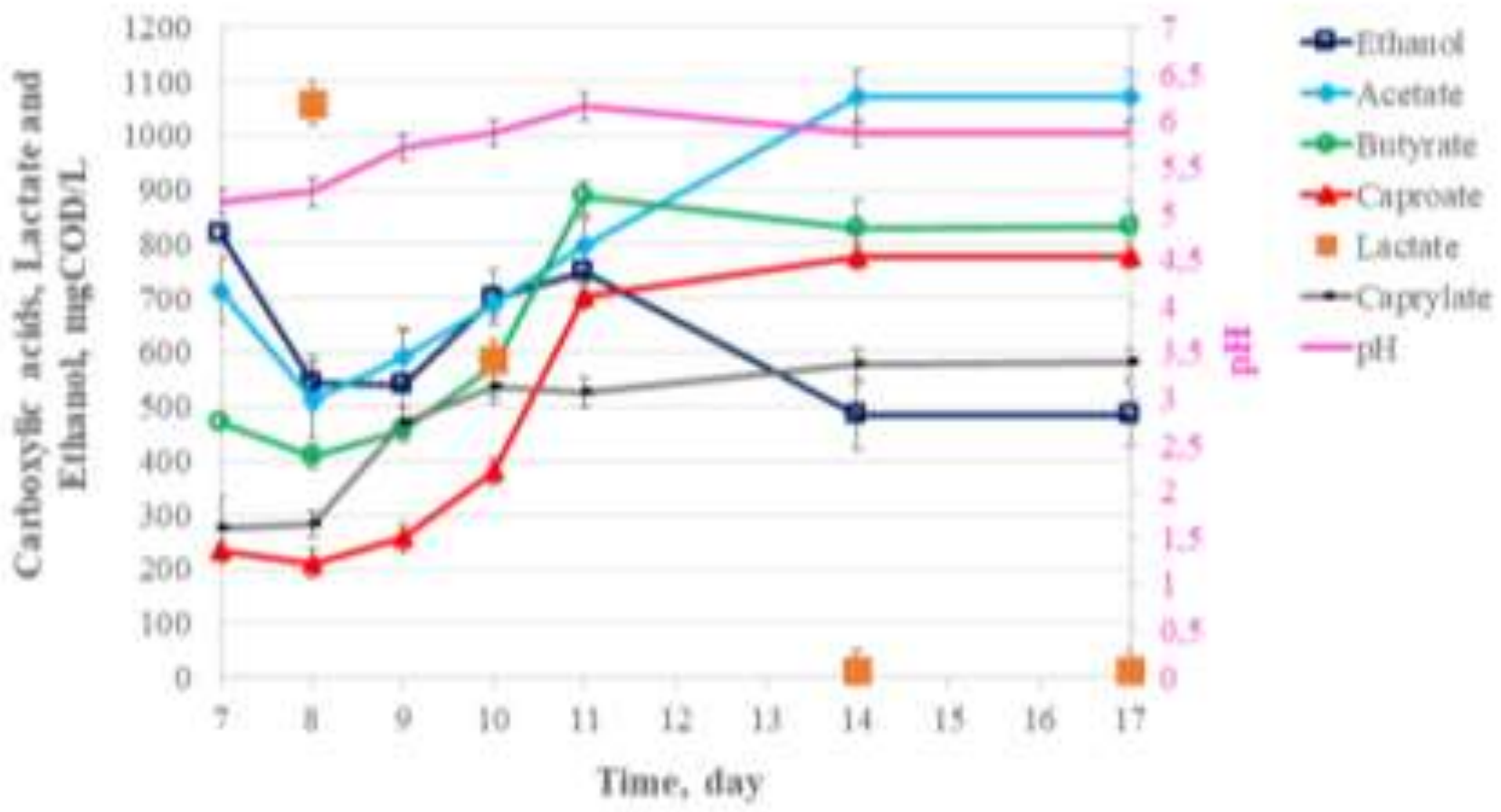

Figure S4. Carboxylic acids production in a) Control and b) Test reactors starting respectively with 1070±4.1 mgCOD/L synthetic lactic acid (ethanol blue square; acetate light blue rhombus; butyrate green circle; caproate red triangle; lactate orange dots; caprylate dark grey dash; pH pink solid line). Error bars represent the standard deviation.

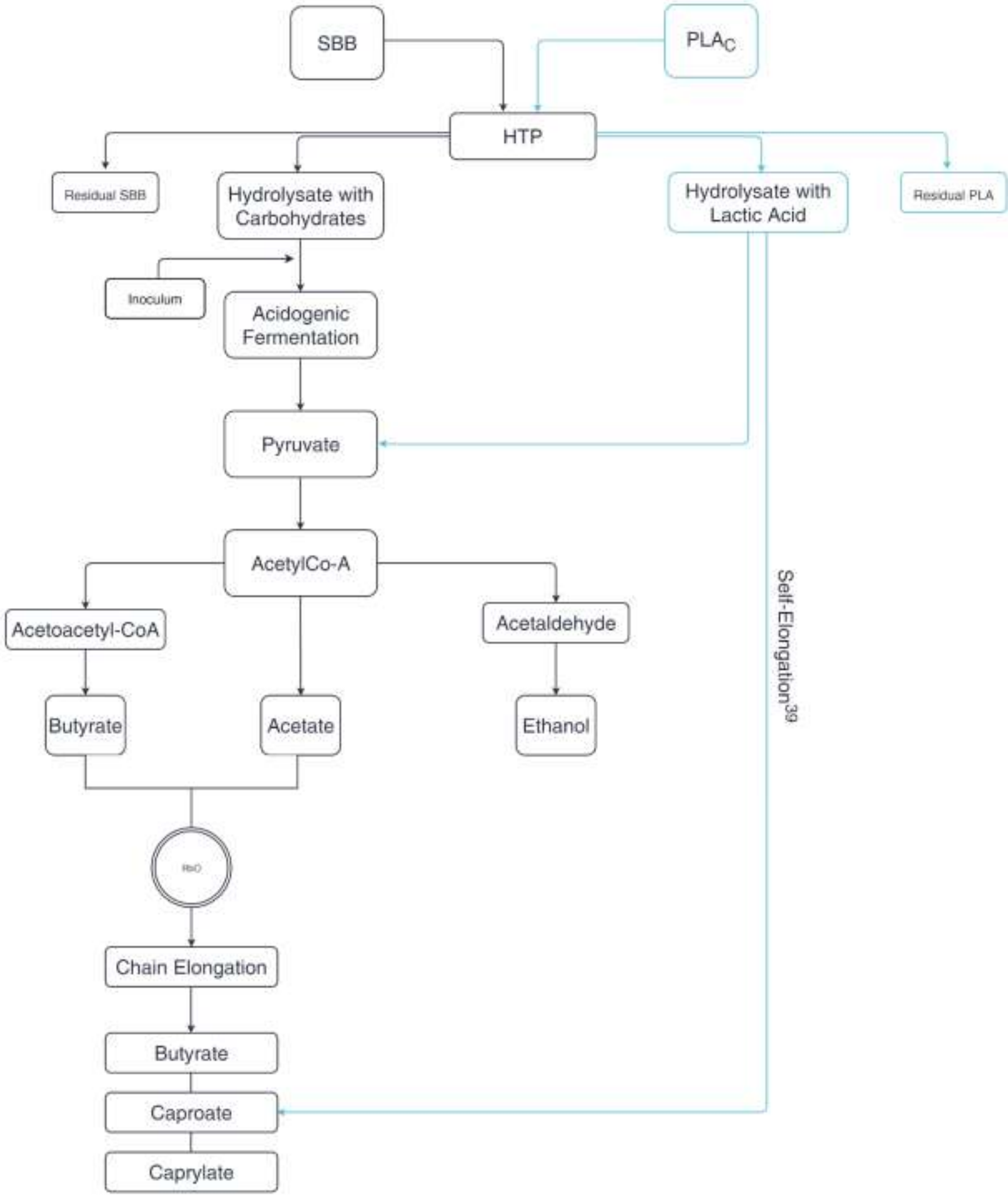

Figure S5. Diagram of metabolic pathways for bioplastics upcycling through acidogenic fermentation and chain elongation.
